# Suppression of Sulfate-Induced Expansion with Lime–Silica Fume Blends

**DOI:** 10.3390/ma15082821

**Published:** 2022-04-12

**Authors:** Mansour Ebailila, John Kinuthia, Jonathan Oti

**Affiliations:** 1 Department of Civil Engineering, Faculty of Engineering, Bani Waleed University, Bani Waleed, Libya; 2 School of Engineering, Faculty of Computing, Engineering and Science, University of South Wales, Pontypridd CF37 1DL, UK; john.kinuthia@southwales.ac.uk (J.K.); jonathan.oti@southwales.ac.uk (J.O.)

**Keywords:** sulfate bearing-soil, ettringite, calcium-based stabiliser, lime, silica fume, mechanical strength, linear expansion, swelling

## Abstract

Sulfate-induced expansion resulting from the formation of ettringite in sulfate-bearing soil stabilised with calcium-based stabilisers is a problematic issue with technical and economic implications. Thus, this research examines the viability of the co-addition of lime (L) and silica fume (S) at varying binder dosages (4, 6, and 10 wt%), with a view of establishing the optimum blend of L–S for suppressing the ettringite-induced expansion of artificially high sulfate-dosed soil (kaolinite-K and gypsum-G). To do so, a series of laboratory specimens, designed using different gypsum and lime concentrations, were investigated using unconfined compression strength (UCS), linear expansion, and derivative thermo-gravimetric analysis (DTG) as the main criteria for the examination. The research outcomes indicated that the increasing substitution of L with S induces a gradual reduction on the UCS and linear expansion at binder levels of 4 and 6 wt%, while its usage in a high binder level (10 wt%), can yield an expansion reduction, with no compromise on the UCS performance. Therefore, silica fume has the potential for restricting ettringite formation and suppressing the expansion, of which 3L7S is the optimum blending ratio for suppressing the expansion.

## 1. Introduction

Sulfate soil is encountered in nearly every country in this world and recognised as a problematic soil, due to its devastating repercussion in the presence of calcium-based stabilisers (cement-C and lime-L) [1]. This is due to the formation of ettringite [2], which, in crystallography, possesses a hexagonal prismatic shape and can be ideally expressed with a formula of ${\mathrm{Ca}}_{6}{\left[\mathrm{Al}{\left(\mathrm{OH}\right)}_{6}\right]}_{2}\cdot {\left({\mathrm{SO}}_{4}\right)}_{3}\cdot 26H_{2}O$ [3,4]. Ettringite forms due to the reaction between the soluble sulfate, calcium (from calcium-based binder), and alumina (from the soil) in the presence of water [5]. It has a highly abnormal water absorption and expansive swelling capacity, causing cracks, and even the disintegration, of stabilised materials [6,7,8]. Apart from the negative repercussion associated with the incorporation of calcium-based stabilisers in sulfate-bearing soils, there are also significant concerns associated with their production, in terms of the non-renewable energy (1.5 tonnes of limestone and clay per one tonne of C), the higher energy consumption of its production (~5000 MJ/tonne for C and ~4000 MJ/tonne for L), the higher carbon dioxide emissions (1000 Kg/tonne for C and 800 Kg/tonne for L) emitted in the atmosphere [6,9,10]. Consequently, the incorporation of industrial by-product materials, which are also known as supplementary cementitious materials (SCMs), have been encouraged [11].

Ground granulated blast-furnace slag (GGBS), a by-product of the steel industry, is categorised as one of the ‘greenest’ of engineering SCMs [6] used for soil stabilisation. GGBS is a latent hydraulic material; thus, it is often activated by cement-C, lime-L, or magnesium oxide-M, of which the latter being preferred. In the context of soil stabilisation, C-, L-, and M-activated GGBS blends have been examined and proven to be an effective stabiliser for (1) suppressing the sulfate-induced expansion [1,12,13,14], (2) producing superior resistance to sodium sulfate attack [15], (3) possessing considerable durability improvements, due to the higher resistance of carbonation and hydration products [16], (4) inducing higher heavy metal immobilisation efficiency [17,18] and lower leachability capabilities [19,20], and (5) yielding higher strength performance (about 2–4 times that of cement) [1,11]. However, the future availability of GGBS has become questionable, particularly in the UK, as the entirety of the quantity produced is consumed in Portland cement blends [21]. Therefore, there have been efforts in this endeavour, in the recent past, to focus on other potentially usable SCMs as alternatives to GGBS for soil stabilisation.

As an alternative SCMs to GGBS, silica fume (S), by virtue of its pozzolanic reactivity, has been recently gaining increasing attention in the field of soil stabilisation [22,23,24,25,26,27,28,29,30,31]. Silica fume is a very fine amorphous (nan-crystalline) silica, produced as a by-product of the smelting process of the reduction of high-purity quartz in the silicon and ferrosilicon alloy industries [30,32]. Silica fume, especially un-densified silica fume, has higher pozzolanic activity, where it yields calcium hydroxide consumption up to four times higher than that of GGBS [33]. Regarding its application in sulfate soil, Wang et al. [22], for example, used ternary blends of C–GGBS–S and reported a reduction in the expansion, from 6% to about zero. Ghorbani et al. [23] reported a considerable swelling reduction of sandy soil (having gypsum content of 25% and silicon dioxide of 23%, with no sign of its aluminium oxide) by use of binary L–S blends. Mousavi [25] investigated the co-effect of different C–S blends on the stabilisation of high plastic clay with a sulfate (${\mathrm{SO}}_{3}$) content of 2.7% and concluded that 6C–2S is the optimum blend, in terms of shear and UCS, with no reported result on the expansion. GhavamShirazi [34] studied the effect of binary blends of L–S on the swelling of high plastic clay with a sulfate content of 0.4% and reported a reduction in swelling from 6% to 0.5%, by use of a binary composition of 3L–10S.

Given the explanation above, silica fume is recognised as a highly pozzolanic material and has the potential for suppressing sulfate-induced expansion. However, the benefit of its utilisation for high sulfate soil has still not been thoroughly researched. As an instance, the optimum silica fume dosage, at which the ettringite-induced expansion in high sulfate/alumina-bearing soils stabilised with different levels of lime is suppressed, has not yet been scientifically established. Therefore, intending to fill the knowledge gap, this current study attempts to further examine the co-effect of L–S formulations and propose the optimum binary L–S blends for restricting the ettringite-induced expansion. To this end, the performance of artificial kaolinite specimens dosed with 0 and 9% of gypsum-G and stabilised with different binder levels (4, 6, and 10 wt%) of L–S formulations, has been investigated in this study using UCS and linear expansion and reinforced by thermo-gravimetric/derivative thermo-gravimetric (TG\DTG) analysis.

## 2. Materials and Methods

### 2.1. Materials

**Figure 1:**
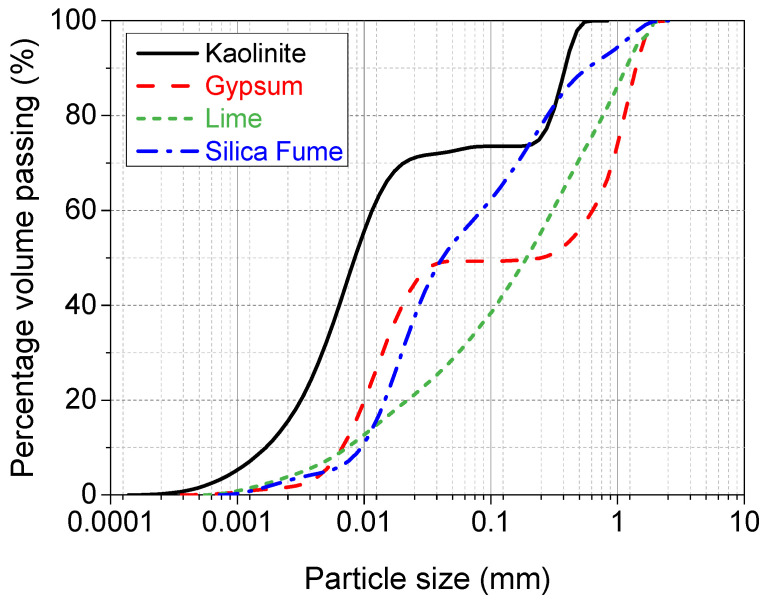
Particle size distribution of kaolinite, gypsum, lime, and silica fume.

**Figure 2:**
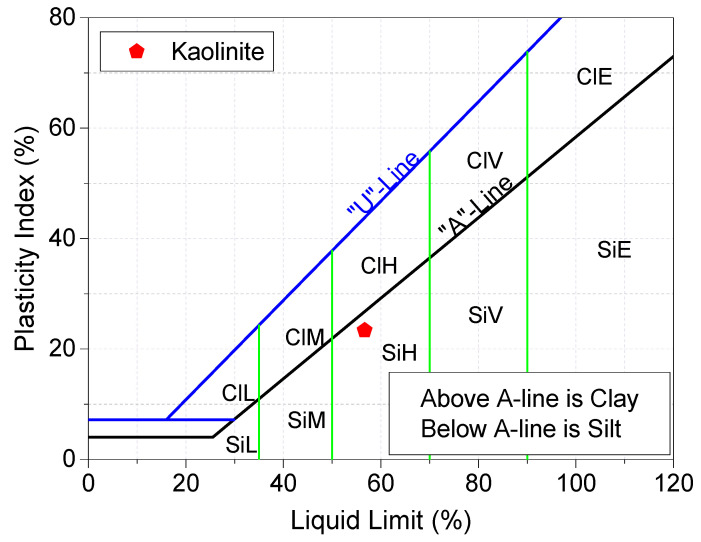
Classification of kaolinite (K), based on the plasticity chart.

The raw materials used throughout this study were kaolinite-K [${\mathrm{Al}}_{2}{\mathrm{Si}}_{2}O_{5}{\left(\mathrm{OH}\right)}_{4}]$, gypsum-G [${\mathrm{CaSO}}_{4}\cdot 2H_{2}O$], lime-L [CaO], silica fume-S [${\mathrm{SiO}}_{2}$, 98.4%], and tap water [$H_{2}O$]. Kaolinite was semi-processed industrial kaolinite, in the form of a fine powder with a relative density of 2.6–2.7, supplied by Potterycrafts Ltd., Stoke-on-Trent, UK, under a commercial trade name of China clay standard porcelain powder. The particle size distribution, shown in Figure 1, suggested that K contains 27% sand, 61% silt, and 12% clay, while the Atterberg limits and plasticity index chart (see Figure 2) showed that it has a liquid limit of 56.7%, plastic limit of 33.3%, and plasticity index of 23.4%. Therefore, as per the grain size and plasticity index classification systems, the kaolinite used is a medium graded sandy SILT of high plasticity.

Gypsum was calcium sulfate dihydrate, in the form of a fine white powder with a percentage purity of ≥98% and water solubility of 2 g/L (20 ℃). It was produced by precipitation from an aqueous solution and obtained from Fisher Scientific Ltd., Loughborough, Leicestershire, Leicester, UK. Lime was quicklime, in the form of an off-white, finely odourless powder with a relative density of 3.31 and supplied under a trading name of lime-base quicklime by Tarmac Cement and Lime Company, Buxton Lime and Powders, Derbyshire, Derby, UK. Silica fume was a commercially highly reactive micro-silica, in the form of a light grey amorphous power with a ${\mathrm{SiO}}_{2}$ content of 98.4%. It was manufactured by Elkem Silicon Materials in Norway [32], and supplied by Tarmac Cement and Lime Company, Buxton Lime and Powders, Derbyshire, Derby, UK, under the trading name of Elkem un-densified micro-silica 971. Figure 1 shows the particle size distribution curves of the raw materials, while Table 1 and Table 2 summarise the oxide compositions and physical properties respectively.

### 2.2. Mix Proportions

Formation of ettringite in lime-stabilised sulfate soil is controlled by several factors, such as clay mineralogy, sulfate content, and binder content, among other variables [35]. Therefore, to conduct a precise study, it was found necessary, at the initial stage of the author’s research on the application of silica fume, to study the effect of a broad range of binder contents for the stabilisation of sulfate soil with a constant sulfate content. In this regard, the preliminary mixes under this current study (see Table 3) were designed using kaolinite (K), two gypsum concentrations (0 and 9 wt%), three binder dosages (4, 6, and 10 wt%), and different lime substitution levels with silica fume. A two-part notation system was used to express the mix code. The first part typifies the target soil materials (K0G or K9G), which represents the acronym form of kaolinite-K containing 0% or 9% of gypsum-G. The second part signifies the acronym form of the binary blend of lime (L) and silica fume (S). The gypsum percentage shown in the mix code represents the gypsum concentration in the target soil material (kaolinite and gypsum), while the binder dosage represents the binder percentage by weight of the target soil materials.

The artificial processed target soil materials (K0G and K9G) were used in this study because of the consistency and homogeneity of kaolinite [36], as it facilitates the ease of identifying and explaining some complex interactions before venturing into more complex natural unprocessed clay soils in future work [37]. The adoption of kaolinite and 9% gypsum stems from the hypothesis that, if the optimisation of binder combination was carried out using the worst sulfate scenario (high gypsum/alumina-enriched blended soil) for ettringite formation, ettringite formation in any other sulfate-bearing soil stabilised with the optimum binders would be expected to be manageable, to some reasonable degree. The three binder levels (4, 6, and 10 wt%), which were within the typical binder range for modification/stabilisation of different soils, were adopted in this study to establish a more general and unbiased platform. This enables the generalisation of the optimised binder and facilitates the speculation of the optimum blended binder for different natural soils. As for the lime replacement with silica fume, it initially occurred at a small replacement interval (10% by dry weight of binder) and then continued at 20% replacement intervals, taking into consideration enough lime content (1–2% by weight of target soil material) has existed for fabric modification and initiation of the pozzolanic reactions. The replacement intervals were found vital to establish the exact optimum silica fume dosage, at which the ettringite-induced expansion in high sulfate-bearing soil stabilised with different lime levels, is suppressed with no compromise on strength.

### 2.3. Sample Preparation

At the introductory stage of laboratory simulations, it was vital to carry out proctor compaction tests, in accordance with [38] and establish the optimum moisture content (OMC) at which the maximum dry density (MDD) is obtained. Due to the cost implication of the proctor compaction test, however, this test was limited to K0G-based mixes, where only the variation of lime levels was investigated. Accordingly, the OMC and MDD were discovered to be 27% and 1455 $\mathrm{kg}/m^{3}$ for K0G-4L0S, 27% and 1440 $\mathrm{kg}/m^{3}$ for K0G-6L0S, and 28% and 1420 $\mathrm{kg}/m^{3}$ for K0G-10L0S, respectively. However, in practice, soil is always compacted at a moisture content (MC) relatively higher than that of OMC [39,40,41], as this actualises the best performance. This is due to the fact that the relatively higher moisture content accommodates any moisture losses during the compaction stage. Therefore, a MC of 30%, equal to 1.1 × OMC, was adopted for the fabrication of testing specimens made with 4 and 6 (wt%) of binder, while 31% was used for 10 wt%. By keeping the MC constant, the specimens were expected, within the experimental error, to be made at comparable densification.

**Figure 3:**
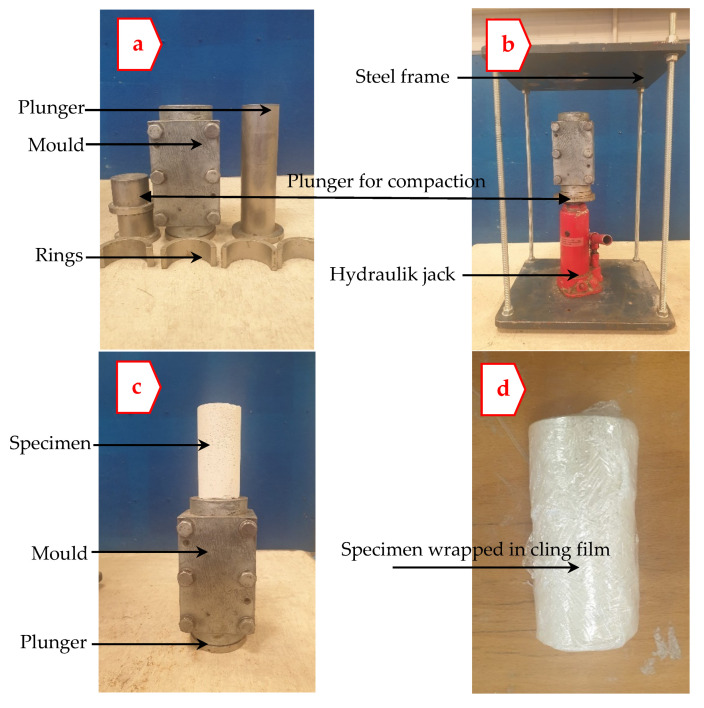
Apparatus used for specimen preparation, (**a**) steel mould to accommodate the semi-paste mixture, (**b**) steel frame and hydraulic jack for specimen compaction, (**c**) extruded specimen and (**d**) extruded specimen wrapped in cling film.

A total of 11 specimens per mix were produced from dried K mixed with G, L, and S at the predetermined MC. For each specimen, enough dry materials capable of producing a specimen (measuring 100 mm in height and 50 mm in diameter) were mixed in a mechanical mixing device for 3 min, before progressively being humidified with the predetermined MC. Afterwards, an intermittent hand mixing with a palette knife, followed by re-mixing of the moistened mixture in the mixing device for a further 3 min, was performed to enhance the homogeneity of the mixture. On completion of mixture homogenisation, the semi-paste mixture was carefully poured into a 100 mm × 50 mm prefabricated cylinder-shaped mould (see Figure 3a) fitted with a collar to accommodate all the materials. The homogenised mixture was then compressed using a hydraulic jack wherein a static compressive force was axially applied in aid of a fabricated custom-built steel frame (see Figure 3b), to achieve the desired dry density. The compacted specimens were therefore kept in the mould for 3 min, enabling specimen stability. Once specimen relaxation was achieved, the specimen was then cautiously extruded using a steel cylinder-shaped plunger pre-lubricated with a thin film of oil to ease the extrusion (see Figure 3c). The prepared specimens were trimmed and individually wrapped in several runs of cling film (see Figure 3d) to regulate humidity and reduce moisture evaporation, complying with [42]. Finally, the cylinders produced were collectively kept in a sealed container to moist cure and stored in a temperature-controlled room of 20 °C until the date of testing.

### 2.4. Testing Method

To evaluate the performance of the designed formulations, three tests, including linear expansion, UCS, and TG/DTG analysis, were adopted in this study.

The linear expansion was carried out on two cylinders per mix composition, in accordance with [43], using perspex cells, such as those employed elsewhere [37,44,45]. The use of replicates for the stabilised specimens was due to expected heterogeneity post-stabilisation and informed confidence in the repeatability and effectiveness of the optimised binary blends, as well as the precision/reliability of the results [46]. On the other hand, the utilisation of the linear expansion method for monitoring the volume change behaviour was because it has several advantages over those of the standardised methods, including swelling pressure, swelling potential, or the California bearing ratio method. Simplicity and availability of an adequate number of perspex cells, in addition to the ease of test operation, are some of the vital reasons wherefores the linear expansion method was selected. The complex nature and high-cost practicability of the scheme, particularly that of the California bearing ratio, was also a contributing factor to the adoption of the linear expansion method.

**Figure 4:**
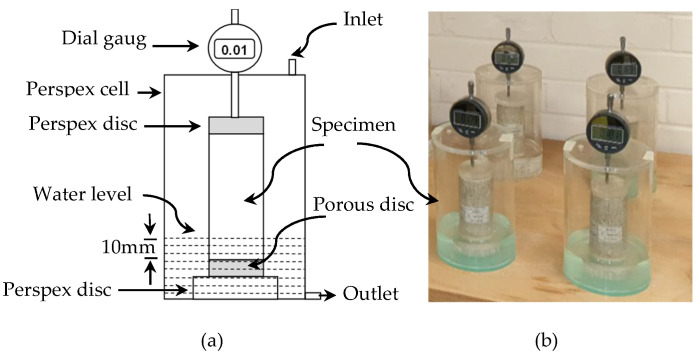
Linear expansion test; (a) schematic diagram of a perspex cell test set-up, (b) specimens during the linear expansion test.

Immediately after 7 days of moist curing, about 10 mm of the top and bottom of two compacted cylindrical specimens, per mix composition, were unwrapped by carefully cutting and removing the cling film using a sharp razor. The partially exposed compacted specimens were then individually placed on a separate porous disc, located on a plastic platform in a perspex cell, as schematically shown in Figure 4. The perspex cells were thereafter covered with prefabricated lids, equipped with dial gauges to measure the vertical displacement (vertical expansion) of the specimens. This was followed by adjustment of the dial gauges, such that the dial gauges are working appropriately and touching the top perspex disc. On completion of the initial reading recordation, the water was carefully added to the perspex cells, through the top inlet, using a siphon to ensure a minimum disturbance of the accommodated cylinders. The level of water was carefully increased, until the exposed bottom part of the specimens (up to 10 mm of the specimen’s base) was completely immersed in water. In addition, the layer of water was always kept constant, at the prescribed level, by adding some water when it was needed, in order to ensure that no water evaporation from the specimens occurred. The process of partial immersion of specimens in water is referred to as soaking [47] and commenced after 7 days of moist curing. The process of soaking was monitored on a daily basis for a period of 200 days.

The UCS was conducted in accordance with [48,49], using a Hounsfield testing machine equipped with a special self-levelling device to ensure uniaxial load application, and it was capable of exerting load up to 10 kN. At the end of each prescribed curing period (7, 28, and 90 days), three-cylindrical specimens, per mix proportion, were unwrapped, weighed, and compared with their original weight at the time of casting. This was conducted to discard any specimens that lose more than 2% of their original mass during the curing period, complying with [48]. The specimens to be tested were immediately mounted into the testing machine, with no drying procedure, and a uniform compressive load, with a constant strain rate of 2 mm per minute, was applied until failure. Ultimately, the mean of the failure loads was used for the establishment of the UCS, based on the ratio of the failure load to the cylinders’ cross-sectional area.

The DTG analysis was performed on randomly oriented portions of powdered pieces of the 7-day UCS specimens, using a TA instruments TGA55 kit. The analysis was conducted from room temperatures up to 1000 ℃, under an argon atmosphere, at a flow heating rate of 20 ℃ per minute. The representative samples used for the analysis were pre-dried in a desiccator at 40 ℃ to ensure moisture equilibrium. Silica gel was also used and replaced every 12 h, to ensure rapid evaporation of moisture, for a total period of 2 days.

## 3. Results

### 3.1. Unconfined Compression Strength

**Figure 5:**
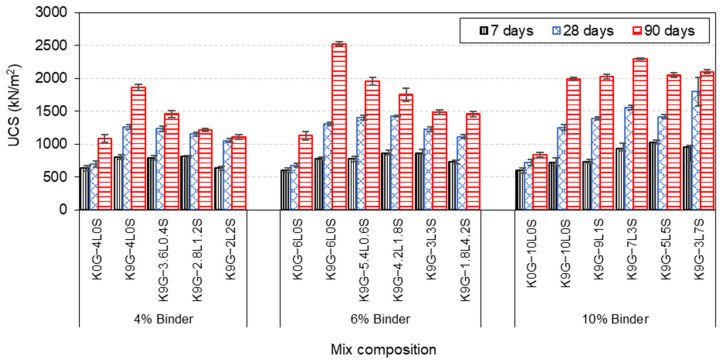
UCS of kaolinite specimens made with various binary blends of lime and silica fume at varying binder dosages (4, 6, and 10 wt%).

Development of UCS with curing times for different kaolinite specimens, designated using various binder levels (4, 6, and 10%), are plotted in Figure 5. In general, all the kaolinite specimens exhibited a progressive development of strength with the curing period, demonstrating the formation of new minerals that can play a significant role in the strengthening of the host matrix. Specifically, at 4 and 6 wt% binder levels, a decreeing strength trend was observed for K9G specimens, in response to the increase of lime substitution level with silica fume, achieving the minimum strength of 1111 and 1459 $\mathrm{kN}/m^{2}$ at 2L2S and 1.8L4.2S, respectively. These lowest strength levels, however, were higher than those of K0G formulations (K0G-4L0S and K0G-6L0S), which experienced a strength value of 1083 and 1129 $\mathrm{kN}/m^{2}$, respectively. As for the high (10 wt%) binder level, the comparative analysis indicated that there was a gradual increase in the UCS, as the lime replacement proportion increased up to 30%, where the strength increased from 1995 to 2027 and 2297 $\mathrm{kN}/m^{2}$ when 10% and 30% of S were used, respectively. Further increase in the substitution level (50% and 70%) yielded a slight reduction (200 $\mathrm{kN}/m^{2}$) in strength improvement, although such strength was greater than those of their counterparts (K0G-10L0S and K9G-10L0S). It is also worth mentioning that, at a constant blending binder ratio (2L2S, 3L3S, and 5L5S), the UCS increases as the lime binder content increases. This implies that it is reasonable to speculate that a higher UCS may be achieved by increasing the binder level, on the basis of 50%L-50%S, such as 6L6S, 7L7S, and so on. Ultimately, the utilisation of silica fume as a lime substitution induces a gradual compromise on the UCS, at both low (4 wt%) and intermediate (6 wt%) binder levels, while its usage in the high (10 wt%) binder level yielded a slight strength improvement, as compared to that of the control (K9G-10L0S).

### 3.2. Linear Expansion

**Figure 6:**
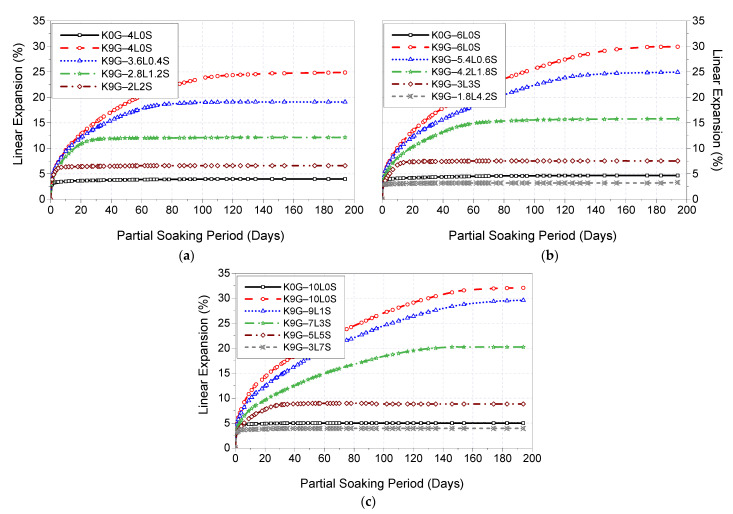
Typical 200-day expansion trends of kaolinite specimens dosed with 0 and 9% of gypsum and stabilised with various binary blends of lime and silica fume at binder dosages of (**a**) 4, (**b**) 6, and (**c**) 10 wt%.

Figure 6 presents the 200-day expansion trends of K0G and K9G specimens stabilised with binary blends of lime and silica fume at different binder dosages (4, 6, and 10%). As shown in Figure 6, the incorporation of silica fume as a lime substitute played a key role in attaining a lower expansion magnitude, and its effectiveness was more pronounced at higher substitution levels. This was presented by a gradual expansion reduction, reaching the lowest expansion of 6.6% for 4% binder specimens, 3.3% for 6% binder specimens, and 3.9% for 10% binder specimens at 50%, 70%, and 70% lime replacement levels, respectively. This indicates signs of the importance of silica fume in restraining the sulfate-induced expansion in lime-stabilised soil. It is also worth mentioning that, at a constant blending binder ratio (2L2S, 3L3S, and 5L5S), the linear expansion increases as the binder content increases, which was expected, due to the increase of lime content in the binder. This also implies that it is reasonable to speculate that the linear expansion is expected to increase gradually, in response to the increase of binder dosage, on the basis of 5L5S (such as 6L6S, 7L7S, and so on).

By keeping the regulatory levels of the 0G-controls in view, it can be also observed that only the L–S ratio of 30%–70%, as shown by K9G-1.8L4.2S and K9G-3L7S, alleviated the expansivity magnitude to a level lower than that of control (K0G). Therefore, it could be concluded that the reduction in the expansivity of sulfate soil stabilised with L–S blends is adversely proportional to the lime content and directly proportional to the silica fume content, of which, an L–S ratio of 30–70 is the optimum for suppressing the expansion in the presence of sulfate.

### 3.3. Derivative Thermo-Gravimetric (DTG) Analysis

**Figure 7:**
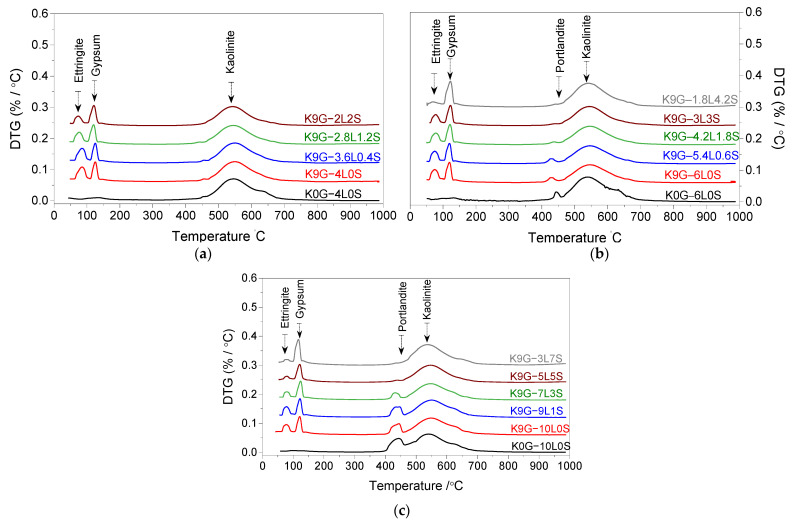
The 7-day DTG curves of kaolinite specimens made with various binary blends of lime and silica fume at three different binder dosages: (**a**) 4, (**b**) 6, and (**c**) 10 wt%.

Figure 7 presents the 7th day DTG curves of sulfate kaolinite soils containing 0 and 9% G and stabilised with different L–S combinations at three binder levels (4, 6, and 10 wt%), alongside the demonstrative comment for the causality of the major peaks. In all binder levels, a progressive reduction in the intensity of the ettringite peak with the increase of silica fume content was evidenced, suggesting a sign of its effect in the mitigation of ettringite formation. This has been substantiated by the regression of the ettringite peak and confirmed by the simultaneous progression of the gypsum peak. With further insight into Figure 7, it can be also observed that the reduction of the ettringite peak is positively proportional to the increase of binder content. Apart from ettringite and gypsum appearance, a shoulder endothermic peak, ascribed to portlandite because of the de-hydroxylation of non-consumed $\mathrm{Ca}\;{\left(\mathrm{OH}\right)}_{2}$ at 400−500 ℃, was also detected in 6L-based specimens and pronounced blunter in 10L-based specimens. This signifies the incomplete consumption of lime during the first 7 days of moist curing. The intensity of portlandite peak was, however, noted to be gradually reduced on the substitution of lime and completely disappeared in the case of intermediate (6%) and high (10%) binder levels, respectively, at an L–S percentage ratio of 70%–30%, as illustrated by K9G–4.2L1.8S and 50%–50%, as given by K9G–5L5S.

## 4. Discussion

### 4.1. Unconfined Compression Strength

The UCS for all the designated formulations exhibited a gradually increasing trend as the curing age increased, providing evidence that stabilised specimens followed the classical trend of soil stabilisation. The display of UCS development is commonly attributed to fabric modification (short-term reaction, including cation exchange and flocculation-agglomeration of soil particles) and pozzolanic reactions (long-term reaction) between soil and lime [50]. In the presence of water, the first reaction taking a place for quicklime is the hydration reaction (an extremely high exothermic reaction), resulting in hydrated lime [35], which further dissociates into calcium and hydroxyl [51]. The released calcium fixes to the surface of soil particles, which causes cation exchange [8,52,53,54], reduces the electrochemical repulsion forces between the soil particles [55], and produces a compressed double cations layer [55,56,57]. As the diffused double cation layer reduces, the electronic-state charges interact to a greater extent, promoting different soil particle arrangements: edge-to-edge flocculation, face-to-edge flocculation, face-to-face aggregation, dispersed-deflocculated fabric, and agglomeration [5,50,58,59,60]. “This process is commonly referred to as flocculation and agglomeration, with flocculation referring to the clustering together of the individual clay particles into ‘‘flocs’’ which in turn agglomerate together into much larger ‘‘aggregates’’ with an open pore structure of reduced packing density” [45].

Besides the release of ${\mathrm{Ca}}^{2+}$, the hydroxides (OH) are also presented, causing an increase in the pH value, up to 12.4 [52,53]. The increase in the alkalinity generates a corrosive environment, in which the alkaline hydrolysis of covalent bonds between Si-O and Al-O releases silicate (${\mathrm{SiO}}_{4}^{-4}$) and aluminate ($\mathrm{Al}{\left[\mathrm{OH}\right]}_{4}^{-}$) [35]. This, therefore, aids the dissolution of aluminate (Al) and silicate (Si) ions, initiating the pozzolanic reactions [6,7], producing cementitious gels CSH and CAH [52,54]. These hydrated products, which are similar to those formed during the hydration of cement [55], crystallise with time, forming a stiff matrix and binding the soil particles by infilling the inter-aggregate pore space [51]. This process, eventually, decreases soil particle movements and enhances strength performance [35].

A comparative analysis of the effect of the sole addition of lime (L) on the UCS performance of kaolinite specimens, in the absence of sulfate, revealed that the binder domination was in the order of 6L > 4L > 10L. The superiority of a moderate lime level (6L) could be credited to the optimum lime content (OLC) that can be consumed during the curing period[54]. Typically, there are two main reactions involved in the consumption of lime: mainly the cation exchange (short-term reaction consumes from 1–3% of lime) and pozzolanic reaction (long-term reaction consumes 2–8% of lime) [61]. Therefore, the addition of a lower amount of lime (4L) resulted in an insignificant strength gain, probably because of the insufficient lime content to both the cation exchange and pozzolanic reaction, which was evident by the disappearance of portlandite peak at 400−500 ℃ in the 7-day DTG curve. Upon the addition of 2% extra (6L), there was enough lime for both reactions, which is probably the reason behind the increase in the UCS of the 6L-based system, relative to the 4L-based system. However, upon further addition of lime (10L), a reduction in UCS was reported, emulating the UCS trends of [62,63]. This decreasing phenomenon is probably due to the presence of unconsumed $\mathrm{Ca}{\left(\mathrm{OH}\right)}_{2}$, as confirmed by the blunter peak of portlandite in the 7-day DTG curve of K0G–10L. According to Chemeda et al. [64], a higher amount of lime leads to the coating of K particles by a layer of adsorbed calcium, which prevents the alkaline attack [65]. This, therefore, reduces the silicon and aluminium released from the soil and delays the pozzolanic reaction. Choobbasti and Kutanaei [66] also urged that the presence of the crystals of portlandite on the surface of soil particles reduces the cohesion between the soil particles and cementitious products.

In the presence of sulfate (${\mathrm{CaSO}}_{4}\cdot 2H_{2}O$), the results indicated that the overall UCS of 9G-kaolinite specimens surpassed those of non-sulfate (0G) kaolinite specimens. This provided robust evidence for the beneficial effect of sulfate and could be assigned to the formation of ettringite, a highly hydrated crystalline mineral, forming due to the reaction between the soluble sulfate, calcium, and alumina in the presence of water [1]. Under moist curing condition (sealed condition), ettringite formation improves strength through three combined mechanisms: (1) the reduction of the porosity of the host matrix, due to the nucleation of ettringite crystals within the pores; (2) the formation of an interlocked matrix, due to growth of ettringite around the soil particles, which helps the soil particles to resist the compressive forces upon the incremental loading; and (3) the dewatering of the system, due to the high water absorption capacity of ettringite, as this increases the dry density [6,7,59]. It is also worth mentioning that the UCS of 9G-based specimens followed the binder domination order of 6L > 10L > 4L. The dominance of 6L, over those of 4L and 10L, can be credited to the threshold of the gypsum/lime (G/L) ratio, in which a decrease or increase in the G/L ratio has no significant contribution to the UCS. This threshold is around a G/L ratio of 1.5, which will be the subject of another publication that reports the result of simulating the strength and expansion of specimens made with varying L and G contents.

Upon the use of silica fume with different lime levels, the result revealed that its dosage of administration yielded two different UCS trends, mainly (1) decreasing and (2) increasing/decreasing trends. The decreasing trend was observed at low (4 wt%) and intermediate (6 wt%) binder levels, in which the inclusion of silica fume induces a gradual compromise on the UCS as the silica fume amount increases. This decreasing trend can be credited to the higher pozzolanic activity of the un-densified silica fume used in this research. According to Suraneni and Weiss [33], the un-densified silica fume used in this research had a pozzolanic activity even higher than GGBS and calcined clay, where it can yield a calcium hydroxide consumption up to four times higher than that of GGBS. This implies that the un-densified silica fume is superior in the acceleration of the consumption of $\mathrm{Ca}{\left(\mathrm{OH}\right)}_{2}$, limiting the available lime for ettringite formation, thus promoting lesser strength gain associated with ettringite. The silica fume also affects the hydration kinetics by promoting more nucleation sites at an earlier hydration age [67], which further accelerates the consumption of $\mathrm{Ca}{\left(\mathrm{OH}\right)}_{2}$. This accelerating phenomenon would then negatively affect the short-term reactions (cation exchange and flocculation-agglomeration of particles); hence, a lower degree of strength gain, associated with fabric modification, would have occurred.

As for the increasing/decreasing trend of UCS, it was observed at a high (10 wt%) binder level, in which the UCS increased to a certain dosage of silica fume, beyond which its further addition induced a gradual reduction in strength. This certain dosage of silica fume was observed by K9G–7L3S, where the K9G system exhibited the threshold of strength at a blending L–S ratio of 70%–30%. The increasing trend (up to the strength threshold) at a high (10 wt%) binder level can be assigned to three factors. The first factor is the formation of much more hydrated products, which induces a pore-blocking effect [40,68] and compensates for the strength reduction induced by the restriction of ettringite formation. The blockage of the capillary pores induces system densification, permeability reduction, and porosity enhancement[45], all of which contribute to strength improvement [61]. The second factor is the provision of a higher binder amount, in the case of 10%, relative to that of 4 and 6%, as this is expected to yield a higher degree of fabric modification. The third factor is the complete consumption of portlandite, particularly at around a 30% lime substitution level, as this leads to overcoming the negative impact of the portlandite between the soil particles and hydrated products, thereby improving the cohesion of the system. As for the decreasing trend (after the strength threshold of 7L3S), this can be assigned to the reduction of lime amount, as this induces lesser hydrated products within the system; thereby, a lower degree of strength gain associated with hydrated products would have occurred. The higher pozzolanic activity of silica fume is also a contributing factor to the decreasing trend, beyond the strength threshold of 7L3S, as it would promote lesser strength gain associated with fabric modification. Eventually, it can be concluded that the UCS of sulfate kaolinite specimens, stabilised with L–S blends, is directly proportional to lime content and adversely proportional to silica fume content, of which, a blend of 7L3S is the optimum for a higher degree of UCS.

### 4.2. Linear Expansion

Prior to stabilisation, soaking of the compacted pure kaolinite specimens in water, after 7 days of moist curing, witnessed a rapid volume enlargement, reaching the ultimate expansion of 6.2% (pre-tabulated in Table 2) at the lapse of the soaking period. The causativeness of the kaolinite expansion is attributed to the inter-crystalline expansion mechanism [69], which is resulted from the enlargement of interparticle pores and wetting of soil particles. Generally, kaolinite clay specimens, in their moist cured state, are a relatively malleable compacted mixture of negatively-charged particles possessing a mineral structure of a leaf-like arrangement and suggesting an interlinked pore structure [70]. On water soaking, water permeates immediately throughout the finer pores of the compacted specimen under capillary suction pressures. Through the upward movement of liquids, the negatively-charged surfaces of kaolinite particles attract the water molecules, altering the internal electrochemical interparticle force equilibrium. An extensive adsorbed film is then formed, owing to the concentration gradient existing between the electrical double-diffused layer and bulk solution, leading to an inter-crystalline spacing change, known as an inter-crystalline expansion. This inter-crystalline phenomenon possesses a rapid increasing nature in the short-term, until a certain degree of saturation, behind which, a steady increasing nature occurs, until the internal electrochemical force system is in equilibrium with the capillarity forces. In this study, the equilibrium of the kaolinite system occurred at an expansion magnitude of 6.2%.

The stabilisation of kaolinite soil (K0G), by use of sole addition of lime, in the absence of sulfate, resulted in a reasonable resistance to expansion. As pre-shown in Figure 6, the descending order of binder predominance by the expansion magnitude was 4L system (4% expansion), 6L system (4.7% expansion), and 10L system (5% expansion). The underlying reaction mechanisms behind lime inclusion were pre-unveiled in the literature, as the result of a short-term (cation exchange, flocculation-agglomeration of soil particles) and long-term (pozzolanic reaction) reactions. The short-term reaction balances the electrostatic charges of soil particles, thus reducing the electrochemical repulsion forces between the particles [55], producing a compressed double cations layer, and subsequently modifying the engineering stabilised soil properties by reducing the water holding capacity of soil particles [56,57]. The pozzolanic reaction produces hydrated products, CSH and CAH, which crystallise with time, forming a stiff matrix [52] and binding the soil particles by infilling the inter-aggregate pore [51] thus enhancing the expansion [35].

In the presence of sulfate, the reference configuration of L-based specimens (K9G–4L0S, K9G–6L0S, and K9G–10L0S) displayed higher expansivity, in which the expansion increased gradually, reaching the ultimate expansion magnitude of 25% for K9G–4L0S, 30% for K9G–6L0S, and 32% for K9G–10L0S at the 120th, 160th, and 180th day of soaking, respectively. Based on the findings of DTG analysis, this increase in the expansion can be associated with the amount of ettringite crystals formed, where the ettringite peak height in both DTG was in line with the expansion trend. Under the soaking condition, ettringite has highly abnormal water absorption, growing as needles or rod-shaped crystals, thereby generating internal stress, causing the formation of cracks and expansion of the stabilised material [6].

Upon the substitution of lime with silica fume, the result revealed that the inclusion of silica fume yielded a decreasing expansion trend at all the binder levels, reaching the lowest expansion value at a blending lime–silica fume ratio of 30%–70%. Similar to the case of UCS, this reduction in expansion is attributed to the restriction of ettringite formation, due to the higher pozzolanic activity of silica fume. The higher pozzolanic activity affects the hydration kinetics by promoting more active nucleation sites for speeding up the consumption of portlandite, thus reducing the solubility rate of alumina. The restriction of alumina dissolution inhibits the formation of ettringite and promotes the formation of CSH over that of CAH, all of which improve expansion.

## 5. Conclusions

The outcome of this research ascertained the feasibility of developing an effective binder using silica fume as a partial substitution of lime, in order to restrict ettringite formation and suppress the expansion associated thereof. The following conclusions can be drawn, as follows.

1. The use of silica fume as a lime substitution at low (4 wt%) and intermediate (6 wt%) binder levels induces a gradual compromise on the UCS as its content increases, while its usage in high (10 wt%) binder level yields a slight UCS improvement, relative to the control (K9G–10L0S). The decreasing phenomenon at binder levels of 4 and 6% can be credited to the faster consumption of lime, as it restricts ettringite formation; thus, the strength gain associated with the growth of ettringite is cancelled. As for the strength gain at 10% binder, this can be assigned to the higher binder amount, as it yields a higher degree of fabric modification and forms much more hydrated products.
2. The expansivity of sulfate kaolinite specimens stabilised with binary blends of L–S is directly proportional to the lime content and adversely proportional to silica fume content, of which, a blending ratio of 30% L–70% S is the optimum for suppressing the sulfate-induced expansion. The decreasing phenomenon, in response to the silica fume increases, is due to the higher pozzolanic activity of silica fume, as it restricts the formation of ettringite, which has been substantiated by a gradual reduction in the broadness of the ettringite peak, coupled with a concomitant increase of the gypsum peak in the DTG curves.
3. Silica fume has the potential to reduce the ettringite formation, but it cannot lead to a complete restriction, probably due to the faster reaction of ettringite.
4. The limitations to this study, which could impact the authenticity of the outcomes, are the utilisation of an artificially sulfate-dosed soil (kaolinite–gypsum) and single silica fume type as the received state (un-densified silica fume without treatment). Therefore, a research study considering the use of different natural soils and pre-treatment of silica fume is recommended to overcome this deficiency.

## Figures and Tables

**Table 1:** Oxide composition of kaolinite, lime, and silica fume.

Oxides	Compositions (%)		
	Kaolinite	Lime	Silica Fume
CaO	<0.01	71.56	0.2
MgO	0.21	0.58	0.1
${\mathrm{SiO}}_{2}$	47.32	0.67	98.4
${\mathrm{Al}}_{2}O_{3}$	35.96	0.07	0.2
${\mathrm{Na}}_{2}O$	0.07	<0.02	
$P_{2}O_{5}$	0.12	0.03	0.03
${\mathrm{Fe}}_{2}O_{3}$	0.69	0.05	0.01
${\mathrm{Mn}}_{2}O_{3}$	0.02	0.02	
$K_{2}O$	1.8	<0.01	0.2
${\mathrm{TiO}}_{2}$	0.02	<0.01	
$V_{2}O_{5}$	<0.01	0.02	
BaO	0.07	<0.01	
${\mathrm{SO}}_{3}$	0.01	0.19	0.1
LOI	0.1	27.4	0.5

**Table 2:** Physical properties of kaolinite, lime, and silica fume.

Physical Properties	Kaolinite	Lime	Silica Fume
$\mathrm{Bulk}\;\mathrm{density}\;\left(\mathrm{kg}/m^{3}\right)$		480	300
$\mathrm{Specific}\;\mathrm{gravity}\;\left(\mathrm{Mg}/m^{3}\right)$	2.14	2.82	3.15
pH Value	5.37	12.62	7
Colour	White	White	Grey
Swelling pressure (kPa)	1.3		
Linear expansion (%)	6.2		
Physical form	Fine powder	Powder	Powder

**Table 3:** Mix composition of artificial kaolinite specimens made with different gypsum concentrations (0 and 9 wt%) and stabilised with binary blends of lima (L) and silica fume (S).

Groups	Mix Code	Lime Substitution Level (%)	Mix Compositions (%)				
			Target Soil Material (%)		Water (%)	Binder (%)	
			Kaolinite	Gypsum		Lime	Silica Fume
4(LS)	K0G–4L0S	0	100		30	4	
	K9G–4L0S	0	91	9		4	
	K9G–3.6L0.4S	10	91	9		3.6	0.4
	K9G–2.8L1.2S	30	91	9		2.8	1.2
	K9G–2L2S	50	91	9		2	2
6(LS)	K0G–6L0S	0	100		30	6	
	K9G–6L0S	0	91	9		6	
	K9G–5.4L0.6S	10	91	9		5.4	0.6
	K9G–4.2L1.8S	30	91	9		4.2	1.8
	K9G–3L3S	50	91	9		3	3
	K9G–1.8L4.2S	70	91	9		1.8	4.2
10(LS)	K0G–10L0S	0	100		31	10	
	K9G–10L0S	0	91	9		10	
	K9G–9L1S	10	91	9		9	1
	K9G–7L3S	30	91	9		7	3
	K9G–5L5S	50	91	9		5	5
	K9G–3L7S	70	91	9		3	7

## Data Availability

Not applicable.
